# Elliptical and linear relationships with rumen temperature support a homeorhetic trajectory for DMI during recovery of feedlot cattle exposed to moderate heat load

**DOI:** 10.1093/jas/skac127

**Published:** 2022-04-13

**Authors:** Megan L Sullivan, Gene Wijffels, A George, Yousef A Al-Hosni, Joseph C W Olm, John B Gaughan

**Affiliations:** 1 School of Agriculture and Food Sciences, The University of Queensland, Gatton, QLD 4343, Australia; 2 CSIRO Agriculture and Food, 306 Carmody Road, St Lucia, QLD 4067, Australia; 3 Data61, CSIRO, 41 Boggo Road, Dutton Park, QLD 4102, Australia; 4 School of Veterinary Science, The University of Queensland, Gatton, QLD 4343, Australia

**Keywords:** feedlot cattle, heat load, physiology, rumen temperature, hyperthermia, respiration rate

## Abstract

Most feedlot animals in Australia experience 2 to 3 moderate heat waves during summer. This study aimed to gain understanding of the physiological drivers in response to and during recovery from such events with a view to designing strategies to ensure rapid and safe recovery. Two hypotheses were tested during thermal challenge and recovery in climate-controlled rooms (CCR): firstly, the feedlot steer on a grain-based diet mounts appropriate physiological responses during moderate heat load and in recovery so that its performance and physiology state after recovery is not different to the feed restricted thermoneutral (FRTN) steer. Secondly, commonly used indicators of increased heat load, e.g., respiration rate (RR), panting score (PS), body surface temperatures (ST), and water consumption (WC), reflect rumen temperature (RT) during thermal challenge and recovery at the level of daily means. In this study, 36 Angus steers (live weight (LW) 451.5 ± 22.6 kg) made up 3 cohorts of 12 animals that sequentially underwent the CCR phase. For this 18-d phase, the steers were allocated to either a moderate heat load treatment (thermally challenged, TC, *n* = 18) or a FRTN treatment (*n* = 18). The TC group underwent 3 periods, Pre-Challenge (4 d, temperature humidity index (THI) range of 68 to 71), Challenge (7 d, THI 73 to 84 with diurnal cycling), and Recovery (7 d, THI 68 to 71). The FRTN group were held at thermoneutral conditions in the CCR (THI 66.9 ± 0.3), and each animal was offered an amount of feed was based on the feed intake of its LW matched TC pair. Thus, as DMI fell in the TC group during Challenge, feed restriction was imposed on the FRTN group. The data were collected by trained observers were DMI, RT, RR, PS, body STs (forehead, shoulder, leg, rump), and WC. Challenge induced a heat stress response in the TC group with reduced DMI and LW, and elevated RT, RR, PS, body STs, and WC (*P <* 0.001). These measures were unchanged or reduced in the FRTN group (*P <* 0.001). At the end of Recovery, the TC and FRTN groups had converged on most measures including LW. Daily mean RT of both groups showed strong linear relationships with THI, RR, PS, head ST, and WC (*P ≤* 0.0022) but opposing elliptical relationships with DMI; that is, as DMI fell with increasing RT for the TC group, DMI increased with rising RT for the FRTN group. In all, the feedlot steers in this study demonstrated sufficient homeorhetic capacity to adjust to moderate heat load and recover from it.

## Lay Summary

Over any summer, most feedlot steers in Australia will encounter 2 to 3 moderate heat waves lasting 5 to 6 d. The ability of these animals to adjust to the hot conditions and recovery from them is not well understood. The advent of obtaining near real-time body (rumen) temperature from feedlot steers has the potential to help feedlot managers, veterinarians and nutritionists make decisions on the handling of feedlots animals during and after heatwaves. Using climate-controlled rooms, this study demonstrated that feedlot steers are physiologically capable of dealing with moderate heatwave conditions. The co-ordinated increases in respiration rate, sweating rate, body surface temperature, and water consumption alongside reduction in feed intake as heat load increases limits the rise in body temperature. By the end of a 7-d recovery period in thermoneutral conditions, the heat stressed steers were not different physiologically and performance-wise from thermoneutral counterparts feeding on a similar amount of food. However, during the recovery period, all animals appeared to be metabolically and physiologically constrained, and did not return to their pre-heatwave feed intake, body temperature or respiration rate. These findings will assist the management of feedlot animals through recovery after a heat wave.

## Teaser

The feedlot steers in this study demonstrated sufficient homeorhetic capacity to adjust to moderate heat load and recover from it. Intensive measure of performance and physiological variables uncovered an elliptical relationship between DMI and rumen temperature which was underpinned by linear relationships between rumen temperature and respiration rate, water consumption and body surface temperature.

## Introduction

The feedlot animal is the most intensively managed ruminant in Australia representing about 4% of the national herd of 25 million cattle at any one time (MLA, 2020, 2021). Most of the feedlots are in regions that experience regular summer heat waves that expose cattle to intervals of high ambient temperatures which puts the animals at risk of heat stress (Howden and Turnpenny, 1997; Trancoso et al., 2020). Moreover, feedlot cattle fed on diets with high levels of metabolizable energy are generally more affected than those in a grazing environment (Brown-Brandl et al., 2003). While attention tends to be directed toward extreme heat wave events that can have fatal outcomes for intensively managed cattle and dairy cows (Lees et al., 2019), most heat waves are not extreme. Based on historical data, the feedlot regions see 2 to 3 heat waves of 5 to 6 d duration and maximum air temperatures of 35 to 36 °C (Trancoso et al., 2020).

The purpose of this study was 2-fold: (1) to ascertain the impact of moderate heat load on the performance and physiology of feedlot steers during thermal challenge and recovery from it, with comparison to thermoneutral steers on a similar plane of nutrition, and (2) to understand the relationships between rumen temperature (RT) with physiological responses commonly used as indicators of increased heat load. By default, the divergent physiological responses of the thermoneutral feed restricted treatment were also captured.

The physiological and performance responses of beef cattle to increased heat loads have been studied in the climate-controlled rooms (CCR) and outdoor pens, thus reductions in feed intake and live weight with concomitant elevations in core temperature and respiration rate are well documented. The advent of the radio-telemetric RT bolus enables core temperature to be continuously recorded regardless of external conditions. In the current study, collection of RT data facilitated detailed assessment its relationships with other variables during moderate thermal challenge and the often-overlooked recovery phase.

## Materials and Methods

This study was conducted with the approval of The University of Queensland animal ethics committee (SAFS/210/13/MLA) in accordance with the guidelines described by the National Health and Medical Research Council (2013) in Australia.

The study was undertaken at the Queensland Animal Science Precinct (QASP), The University of Queensland, Gatton. Thirty-six Black Angus yearling steers were subjected to a regimen that consisted of 4 phases: (1) induction and holding paddock, (2) a feedlot preparation phase, (3) the CCR phase, and (4) a feedlot finishing phase. The feedlot preparation phase (40 d) was designed to manage the feed intake and weight gain of the steers as they adapted to the diet. The feedlot finisher phase (60 d) allowed for continued weight gain and growth before slaughter. Animals were exposed to the natural environmental conditions of a southern Queensland sub-tropical autumn through to winter during the feedlot preparation stage.

The CCR phase was conducted using 3 sequential cohorts of 12 animals (*n* = 36). Animals within a cohort were housed in a group of 12 in the feedlot preparation, CCR and finishing phases. For each cohort, the steers were allocated to a thermal challenge treatment group (TC) or feed restricted thermoneutral (FRTN) group with 6 animals per treatment group per cohort (TC, *n* = 18; FRTN, *n* = 18) for the CCR phase. Only data from the CCR phase (apart from LW change) are presented here.

### Animal management

Cattle were purchased from a commercial farm located in the Northern Tablelands district of New South Wales and transported approximately 300 km (4 h) by road to QASP. The district is an elevated plateau (980 m above mean sea level) with a temperate climate. In the 4 mo before transport the cattle were exposed to maximum air temperature range of 21 to 27 °C, and a minimum range of 12 to 14 °C. The cattle were not exposed to any heat stress before the commencement of the study. Upon arrival at QASP, all cattle were rested for 24 h in a shaded holding pen (12 m × 12 m), with ad libitum access to water and grass hay. After 24 h, the cattle were housed in an adjacent paddock (86 ha) with free grazing on predominately Rhodes grass (*Chloris gayana*) pasture. The paddock was well shaded, and cattle had ad libitum access to water. The cattle remained in the paddock until feedlot entry. No grain or supplements of any nature were supplied to the animals while on pasture. Mean LW (±SD) on arrival was 373.9 ± 30.4 kg/head. For the study, the 36 animals were selected based on breed (Black Angus), age (0 to 2 teeth), sex (castrated male), and temperament (quiet with small flight zone). The use of teeth to age cattle is a common practice within the Australian beef industry, where the number of permanent incisor teeth are used to indicate an age range. Two teeth (i.e., 2 permanent incisors) erupt in *Bos taurus* cattle at an average age 24 mo of age (range 21 to 27 mo of age; MLA, 2017). Flight zone has been assessed as a measure of temperament and efficiency in cattle within our faculty (Parra et al., 2019).

Fifteen days after arrival, all cattle were vaccinated with a 5-in-1 vaccine for clostridial diseases (enterotoxaemia, tetanus, Black disease, malignant edema, and blackleg (Pfizer Animal Health, Australia)), trivalent tick fever (3 pathogens; *Babesia bovis*, *Babesia bigemina*, and *Anaplasma centrale*; Department of Agriculture, Fisheries and Forestry (Biosecurity Queensland), Australia) and were also treated for internal and external parasites (Cydectin, 5 g/L moxidectin solvent, 150 g/L hydrocarbon liquid; Fort Dodge Australia, Australia). The cattle were vaccinated for bovine respiratory disease (Bovillis MH, inactivated *Mannheimia haemolytica*; Coopers Animal Health, Australia) and a second dose of the 5-in-1 vaccine 39 d later. The cattle were administered the second dose of the bovine respiratory disease vaccine and treated for internal and external parasites (as above) 21 d after the first 5-in-1 vaccination. Hormonal growth promotants were not used in the study.

Weather data for this period (16 April to 15 May 2014) were extracted from the record collected by the (Australian) Bureau of Meteorology Gatton weather station (Station identification number 040082; University of Queensland Gatton, latitude 27.54°S, longitude 152.34°E; http://www.bom.gov.au/jsp/ncc/cdio/weatherData/av?p_nccObsCode=122&p_display_type=dailyDataFile&p_startYear=2014&p_c=-321412158&p_stn_num=040082). The weather conditions for 16 May to 1 September 2014 were recorded by an automated weather station (GRWS100 Weather Station, Campbell Scientific, Logan, UT) sited at the feedlot pens. The distance between the 2 weather stations was approximately 2 km. Note that the grazing paddock, feedlot pens, and CCR were all located on the QASP site within 1 km of each other. During the induction and paddock grazing phase, the steers experienced typical autumn weather conditions for this region: daily mean (±SD) minimum and maximum TA of 12.7 ± 3.5 and 26.1 ± 2.9 °C, respectively; daily mean minimum and maximum THI were 55.4 ± 5.1 and 72.8 ± 3.7, respectively.

### Feedlot preparation phase

This phase allowed the cattle to transition from a pasture diet to finisher diet and facilitated management of feed intake and live weight gain before entry to the CCR. The feedlot pens were situated in a north–south alignment with a soil surfaced pen area of 162 m^2^ (27 m × 6 m). There was a 2% slope from the front of the pen to the rear of the pens. Concrete feed bunks with a 3 m concrete apron were located at the front of each pen (western side). Each feed bunk provided a linear area of 0.35 m/animal and the linear water trough area was 0.09 m/animal. Stocking density was 13.5 m^2^/animal. Shade aligned north–south was provided by shade-cloth (black, 90% solar block, Darling Downs Tarpaulins, Toowoomba, QLD, Australia) attached to a 4-m high structure. The shade structure provided a shade footprint of 14.4 m^2^ so that at midday there was 1.2 m^2^ shade/animal.

The commencement of the 40-d feedlot preparation phase was staggered according to cohort (i.e., cohort 1 through to 3 in numerical order) to ensure that all steers were subject to the same management and feeding regimes. Cohorts were balanced by LW, temperament (flight zone) and age (based on teeth). The cattle entered the feedlot during June and July (winter). Cohort 2 entered the feedlot 13 d after cohort 1, and cohort 3 entered the feedlot 21 d after cohort 2. The mean initial non-fasted feedlot entry LW (±SD) for cohorts 1, 2, and 3 were 443.0 ± 17.9, 453.8 ± 20.9, and 457.7 ± 27.2 kg, respectively (*P* = 0.262). Over the entire feedlot preparation phase for all 3 cohorts, the mean (±SD) daily minimum and maximum TA were 7.7 ± 3.0 and 21.4 ± 2.4 °C, respectively, and the respective mean (±SD) daily minimum and maximum THI were 46.9 ± 5.0 and 66.4 ± 2.7. Further information on the specific weather conditions for each cohort while in the holding paddock and during the 40-d feedlot preparation phase is given in Supplementary Table S1.

Cattle within a cohort entered the feedlot on day 1, commencing on a starter diet through to day 16, then transitioned to a finisher diet over 10 d and were the fed this diet for the remainder of the study. Diet compositions are given in Table 1. Cattle were fed twice daily at 0700 and 1630 hours when housed in the feedlot with 50% of the daily ration provided at each feeding. Refusals were removed once daily 30 min before the 0700-hour feeding and weighed. DM was not determined for daily refusals at time of collection and was based on DM value of diet fed. The amount of feed offered daily was determined by the previous day’s pen intake (12 steers) and the amount offered was increased based on the cattle consuming all feed offered for 2 d and a step/hold procedure (increase 1 day and hold amount fed for 1 day, increase following day if animal intake permitted).

**Table 1.** Diet formulation and nutrient composition

Item	Starter	Finisher	Pasture grass mix chaff
*Ingredient, DM% of diet*			
Barley	15.00	25.70	—
Sorghum	35.00	—	—
Wheat	—	30.00	—
Millrun	10.00	10.00	—
Cottonseed meal	2.50	—	—
Molasses	2.00	3.00	—
Limestone	1.30	1.35	—
Sodium bicarbonate	0.80	1.25	—
Urea	0.70	0.67	—
Sulphur (dusting)	0.03	0.03	—
Moneco 200^1^	0.01	0.01	—
Sodium bentonite	2.50	2.50	—
Mineral–vitamin supplement^2^	0.10	0.10	—
Chickpea shell	30.00	25.00	—
*Nutrient composition (DM basis)*			
DM,%	89.70	90.40	89.80
NE_g_, MJ/kg	5.50	5.50	2.20
Crude fat,%	2.90	1.60	2.30
CP,%	15.70	15.80	16.10
NDF,%	22.00	16.90	52.90
ME, MJ/kg	13.10	13.10	8.90

Contained 200 g/kg monensin sodium (International Animal Health, Huntingwood, NSW, Australia) and provided 20 mg/kg of monensin sodium to the final diet.

Contained (on a DM basis): 8,000 IU/g of vitamin A; 2,000 IU/g of vitamin D; 16,000 mg/kg of vitamin E; 12,000 mg/kg of copper; 400 mg/kg of selenium; 200 mg/kg of cobalt; 1,000 mg/kg of iodine; 10,000 mg/kg iron; 50,000 mg/kg of zinc; 30,000 mg/kg of manganese; and 15,000 mg/kg antioxidant.

Non-fasted LW was obtained at 0700 hours once weekly on the same day of the week using calibrated scales and load beams (Gallagher, Australia) mounted under a cattle crush/squeeze chute (Warwick Cattle Crush, Australia). The 0700-hour daily feeding was delayed until after the live weight data collection was conducted on this day each week to prevent consumption of feed immediately before weighing. To conduct the weighing procedure, the cattle were walked from their pen as a group of 12 for a distance of 100 to 200 m depending on feedlot pen location to a handling facility. There was no feed or water curfew prior to weighing. The scales used to measure live weight were calibrated before commencement of the trial.

### Climate-controlled room phase

The CCR had 4 rooms with 3 pens per room. The rooms are configured in pairs such that one room can be set to thermoneutral conditions and the other to the thermal challenge conditions. The pens (steel frame) were 2.5 m × 2.5 m with rubber mat flooring (RPS Industries, Australia) over a steel grill to allow for cleaning (daily at 0830 hours), drainage from pens and provided a softer surface for the steers. Within a room the cattle could see and hear each other but they were not able to make physical contact with one another. Each animal had individual access to a water trough and a feed trough. A feed trough (500 mm long × 500 mm wide × 500 mm deep) and a water trough (500 mm long × 500 mm wide × 500 mm deep) were located at the front of each pen. The water level in the water trough was maintained at 300 mm to reduce the opportunity for cattle to splash water out of the trough. A metal sheet attached to the top of the trough limited cattle access to 400 mm of the length. Again, this was to reduce the opportunity for cattle to splash water out. Cattle could still place their whole head in the water trough. The lighting schedule was 10% lighting from 1900 to 0530 hours, and 100% lighting outside of these hours.

Each cohort of 12 animals was housed in CCR for an 18-d period. Cohort 1 entered the CCR on 27 July 2014, cohort 2 on 11 August 2014 and cohort 3 on 1 September 2014. CCR entry day mean LW (±SD) for cohorts 1, 2, and 3 were 511.2 ± 24.1, 526.0 ± 24.1, and 520.5 ± 22.4 kg/steer, respectively, and were not significantly different (*P* = 0.310). The cattle entered the CCR with an average intake of 10.85 kg DM∙steer^−1^∙d^−1^ of grain (finisher diet) and 1.08 kg DM∙steer^−1^∙d^−1^ of chaff (pasture grass mix ^−^ predominantly (80%) Rhodes grass – *Chloris gayana* with 20% pasture grass mix). The chaff was added to the ration for the CCR phase to improve the fiber content of the ration as part of the Australian feedlot industry recommended practice for feeding feedlot cattle under heat load conditions. The cattle were fed once daily at 0700 hours with 100% of the daily ration provided at this time. The amount of finisher diet offered was based on the previous day’s refusals (i.e., increase/decrease dependant on refusal amount). The amount of chaff offered remained at 1.08 kg DM throughout the CCR phase. Days −1, 0, and 1 in the CCR were an acclimatization period which allowed the animals to become familiar with the new housing, feed regimen, and climatic conditions (data were not collected during this period).

Within each cohort, animals were allocated to a pair based on LW (animals in a pair had a maximum LW difference of 40 kg at allocation) with a pair consisting of 1 TC treatment animal and 1 FRTN treatment animal. The CCR entry day mean (±SD) LW of the steers allocated to the TC treatment was not different to that of the steers allocated to the FRTN treatment (*P* = 0.783; TC: 522.8 ± 24.3 kg/steer; FRTN: 523.7 ± 22.6 kg/steer). In the CCR phase, the TC animals were offered the amount of feed based on its previous day’s intake, i.e., the previous day’s intake plus 20%. The FRTN animal was then offered the same amount on same day. For example, if a TC animal ate 5 kg on day 6, it was offered 6 kg (5 kg + 20%) on day 7, and their FRTN counterpart was given 6 kg on day 7 also. This ‘pair offering’ approach is an alternative to the pair feeding regime used by O’Brien et al. (2010). The method used in the current study should overcome the confounding effects of dissimilar planes of nutrition without inducing the stress of a sudden reduction in feed intake likely to be experienced by the FRTN animals. However, there were 3 pairs (of the 18 pairs) where the TC animal underwent large sudden reductions of intake in the first days of Challenge (days 5 to 7); in these cases, the amount offered to the FRTN animal was stepped down more gradually over 2 d until it could be given the same amount as its TC counterpart (usually by days 7 to 9).

### Thermal conditions

Climatic conditions in the CCR were controlled by a heat exchange system which allowed TA and RH ranges to be set. There were 100% air exchange 24 times/h. Climatic conditions were monitored using TA and RH data loggers (HOBO UX100-011, Onset, MA) every 10 min. The TA and RH data were used to calculate the temperature humidity index (THI), where THI = (0.8 × TA) + {[(RH/100) × (TA − 14.3)] + 46.3} (NOAA, 1976)). The THI is a derived statistic (Thom, 1959), which has been adapted over time to be the de facto heat stress indicator in livestock (Gaughan et al., 2012). The THI formula applied here is the basis for the Livestock Weather Safety Index (LCI, 1970) and is used by the U.S. National Weather Service for advisories (USDC-ESSA, 1970): Normal, ≤74; Alert, 75-78; Danger, 79-83; Emergency, ≥84.

**Figure 1.**
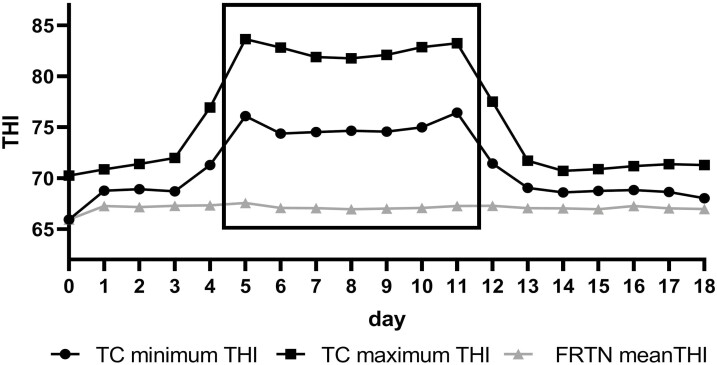
Temperature-humidity indices (THI) for the thermally challenged (TC) group and the feed restricted thermoneutral (FRTN) group during the 18-d trial in the climate-controlled rooms. (A) Daily maximum and minimum THI imposed on the TC group, and the daily mean THI experienced by the FRTN group. The boxed area denotes the Challenge period.

The THI for both treatments is presented in Figure 1. The FRTN treatment were maintained in stable thermoneutral conditions through the 18 d in the CCR: TA, 20.34 ± 0.31 °C; RH, 71.51 ± 3.27%; THI, 67.20 ± 0.46 (mean ± SD; Figure 1 and Supplementary Table S2). Thus, the FRTN animals were maintained under the ‘Normal’ THI category for the entire experiment. The TC group experienced diurnal cycling of TA, RH, and THI. The TC treatment consisted of 3 periods. Days 5 to 11 imposed Challenge thermal conditions on the TC animals, that is, 7 d with THI 74 to 83; while Pre-Challenge (days 0 to 4) and Recovery conditions (days 12 to 18) were at thermoneutral conditions, that is, 4 d at THI 66 to 72, and 7 d at THI 68 to 72, respectively (Figure 1). Days 4 and 12 are considered transition days. In terms of THI categories, the TC treatment animals were maintained under the ‘Normal’ THI category in Pre-Challenge and Recovery, under the ‘Danger’ THI category in the Challenge period for daytime hours and ‘Aware’ category for the night-time hours. The daily maximum, minimum, and mean values for TA, RH, and THI for the TC treatment are presented in Supplementary Table S2.

To model a natural circadian pattern each day during the Challenge period, the CCR were programmed to deliver hourly increments in TA and RH from 0700 hours so that the daily maximum was achieved at 0900 hours, and prevailed till 1600 hours. Both TA and RH then decreased hourly from 1600 hours to reach the daily minimum at 2000 hours. Pre-Challenge and Recovery days were maintained under thermoneutral conditions.

### Physiological data collection

Physiological data were collected on days 1 to 18 during the CCR phase. Data were collected at 2 h intervals between 0600 and 1800 hours each day, and every 2 h over a 24-h period during Challenge by observers for both treatment groups. At each data collection time point, the following measures were collected: respiration rate (RR), body surface temperature (ST) (head, shoulder, rump and lower leg), and water consumption (WC). Daily as-fed feed intake was collected (subsequently converted to DMI) by removing refusals daily 30 min before feeding, weighing the refusals and subtracting this value from the allocated feed delivery amount of the day prior. LW was obtained using calibrated scales and load beams (Gallagher, Australia) mounted under a cattle restraint crush/squeeze chute (Warwick Cattle Crush, Australia) on 10 occasions at 0700 hours (entry and exit to CCR and d 2, 5, 7, 9, 10, 12, 14, and 16) when housed in CCR. Animals were walked 5 to 20 m depending on individual pen location to the crush/squeeze chute for live weight measurements. Respiration rate (RR) was determined by measuring the time taken for the animal to take 10 breaths as determined by flank movement. This value was then converted into breaths per minute (bpm).

Measurement of body STs, as opposed to skin temperatures, is a potentially rapid, inexpensive, and simple means of obtaining a proxy for body temperature in production animals (Church et al., 2014; McManus et al., 2016). As such ST measurement would be highly transferable to the animal production industries. Surface temperature was measured using an infrared thermometer with an accuracy of ±1.0 °C and resolution of 0.1 °C (TN410LCE, Zytemp, Taiwan, China) at a distance of 1 m, with all measurements taken on the left side of the animal. Four sites were assessed. The measurement sites were not shaved. A painted circle indicating that the area to measure the temperature was used to ensure consistency was maintained in measurement sites. The shoulder ST was obtained centrally on the deltoideus muscle. The rump ST was obtained centrally on the middle gluteal muscle. The lower leg ST was obtained halfway between the hock and fetlock joint (metatarsus) on the leg. The steers’ head ST was obtained on the dorsal surface at the midpoint between the left and right eyes. Measurement sites were free of debris and manure when measurements were taken.

Unchilled water was provided ad libitum, and WC was recorded manually at each observation period using water meters (RMC Zenner, Australia). The water trough was located outside of the pen which restricted the animal’s ability to ‘play’ with the water as such. The steers were observed to dip their heads into the water occasionally but most of the water dripped back into the trough as they tend to keep their head over the trough. There was no significant water lost this way. Video cameras were set up in each room to allow for 24 h observation of the animals as required by animal welfare guidelines. At the cessation of the Challenge period, the 12 animals of the cohort 1 were removed for slaughter and post-mortem tissue samples harvested to assess the effects of Challenge at the tissue and cellular level (data not reported here).

### Rumen temperature

Internal body temperature was obtained using rumen dwelling temperature boluses (model SSL001-60, SmartStock, Pawnee, OK) that were orally administered at entry to the feedlot preparation phase using a customized balling gun (SmartStock). Individual boluses were cylindrical in shape (3.3 cm diameter × 8.3 cm in length) and weighed approximately 114 g. The temperature resolution and range were 0.056 and 30.5 to 44.4 °C (SmartStock). Prior to administration the boluses were placed in a 39 °C water bath for 24 h and calibrated to a National Institute of Standards and Technology certified thermometer (model ACC1003FC, range −1 to +51 °C, ThermCo Products, Lafayette, NJ). The boluses contained an active radio-frequency identification transmitter operating within 915 to 928 MHz frequencies range. The radio transmissions were communicated via yagi antenna to a base station and transcribed to a database using proprietary software (TechTrol Inc., Pawnee, OK). Rumen temperatures were transmitted and recorded every 10 min. At each data transmission, the previous 11 data points (110 min) were also transmitted to the database, thereby minimizing the potential for data losses.

RT recordings with a value of ≤35 °C collected but were omitted from downstream analysis. Large WC events (≥8 L) of chilled or cool water are known to reduce RT markedly and rapidly; however, the magnitude of the fall and the time taken to return to pre-consumption RT can be minutes to hours (Brod et al., 1982; Bewley et al., 2008; Ammer et al., 2016; Cantor et al., 2018). There is no formally or generally accepted lower RT threshold or WC time window for sanitizing the RT readings impacted by WC events. Relevant to this study are the observations from Cantor et al. (2018) that ingestion in the range of 6 or 11 L of water at 16 or 29 °C by mature dairy cows was associated with falls in the order of 1.5 to 4.5 °C in RT, which recovered to baseline within 8 to 35 min.

### Statistical analysis

Physiological measurements (RR, RT and ST) were converted to daily and hourly means for each animal. Mean daily physiological measurements were analyzed using a repeated measures ANOVA with an auto-regressive error structure (SAS MIXED procedure) model including animal, pen, cohort, day, treatment, treatment × day, and treatment × hour of day. Effects were estimated for treatment, day, and hour. Where day/hour effects were significant (*P* < 0.05), pair-wise comparisons of means were carried out. For RT measurements, a repeated measures model with a first-order auto-regressive error structure (SAS) was used. The model included pen, cohort, treatment, day, hour, and animal.

For DMI and WC, a repeated measures model with a first-order ante-dependence covariance structure (SAS) was used. The model was selected on the basis of the Akaike Information Criterion and fitted using the SAS MIXED procedure. Denominator degrees of freedom for statistical tests were calculated using the Kenward–Roger method. For LW, the GLM procedure (SAS) was used. The model included cohort and treatment.

Simple linear regression conducted in Prism 9.0 (GraphPad Software, San Diego, CA) was used to discover and describe relationships between variables including the daily means over the 18 d in the CCR, and the hourly means during Challenge and Recovery. *P*-values of <0.05 were considered significant and tendency toward significance was mentioned when *P*-values ranged from 0.05 to 0.08.

Ellipses were fitted to the mean daily RT and DMI data as follows. Consider *y* to be a random variable denoting DMI in kg∙steer^−1^∙d^−1^, and *x* to be a random variable denoting RT (°C). Then the general equation for a rotated and translated ellipse is


$$\begin{array}{l} ax^{2}+bxy+cy^{2}+dx+ey+f=0 \end{array}$$

where *a*, *b*, *c*, *d*, *e*, and *f* are parameters requiring estimation. The parameters were estimated via the best-fit method of least squares, as implemented in the R package, MyEllipseFit (Loewe 2017). This package computes parameter estimates and the goodness-of-fit measure, *R*^2^.

## Results

### Dry matter intake

**Figure 2.**
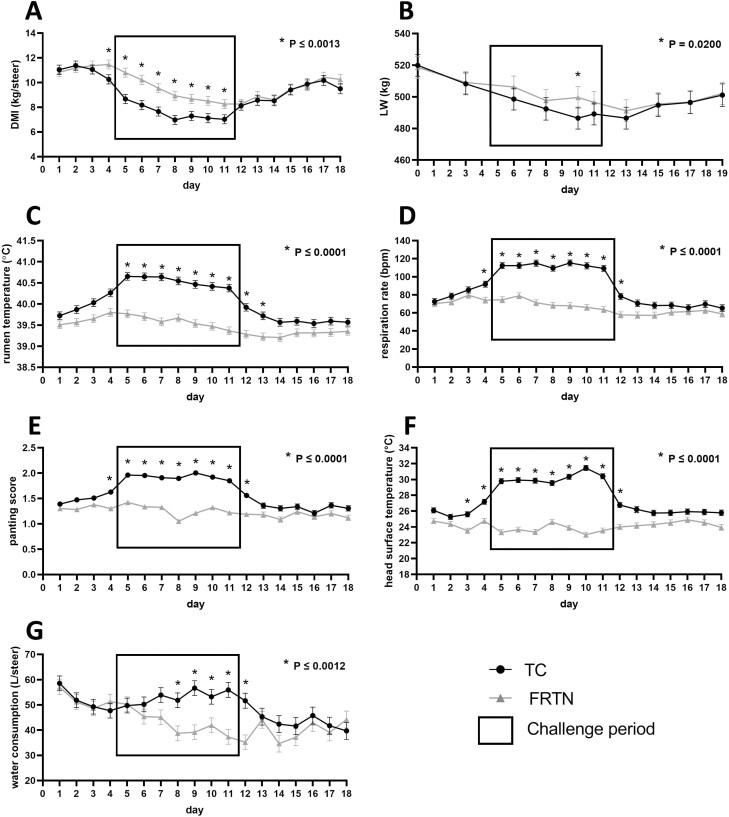
Animal performance and physiological responses of the thermally challenged (TC) and feed restricted thermoneutral (FRTN) groups during the 18 d in the climate-controlled rooms. The daily mean (±SEM) for each measure and each treatment group are presented. The boxed area indicates the Challenge period. (A) DMI. (B) Live weight (LW). (C) Rumen temperature. (D) Respiration rate. (E). Panting score. (F) Head surface temperature. (G) Water consumption.

The decline of mean daily DMI by the FRTN animals during the Challenge period reflected the restricted feeding imposed on these animals (Figure 2A). Minimum DMI for this group was achieved on day 11 at 8.26 kg∙steer^−1^∙d^−1^ with a steady rate in reduction of DMI during Challenge of approximately 0.42 kg∙steer^−1^∙d^−1^. Mean daily DMI of TC animals decreased from day 4 and was on average, 1.7 kg∙steer^−1^∙d^−1^ less than FRTN animals for all days during the Challenge period (*P* ≤ 0.001; Figure 2A). Differences in DMI between treatments for Challenge were due to FRTN animals consuming most if not all feed offered, whereas the TC animals did not. The TC group underwent an initial fall in DMI at a rate of 0.56 kg∙steer^−1^∙d^−1^ over days 5 to 8 arriving at the minimum DMI of 7.0 kg∙steer^−1^∙d^−1^. This was followed by stable DMI at 7.1 kg∙steer^−1^∙d^−1^ for the remainder of Challenge (days 8 to 11). From day 12, as Challenge conditions abated, the animals of both groups increased DMI and consumed all feed offered such that in Recovery (days 12 to 17) the mean daily DMI of both treatment groups were similar (*P* > 0.05); and the rate of refeeding was 0.4 kg∙steer^−-1^∙d^−1^ (Figure 2A). Over days 16 to 18, DMI for both groups appeared to arrive at new level that was approximately 12% less than that of the Pre-Challenge DMI.

### Live weight

Changes in LW of TC and FRTN steers were similar (Figure 2B). During Challenge, there was LW loss in both treatments due to reductions in DMI, either as a response to increased thermal load or restricted feeding. The TC animals entered the CCR at 519.9 ± 6.9 kg and exited at 501.1 ± 7.1 kg; FRTN animals entered the CCR at 518.6 ± 6.9 kg and exited at 502.1 ± 7.1 kg. There was significant difference in LW on day 10 only (*P* = 0.020) at which point the FRTN mean LW was 13.1 kg/steer or 2.7% heavier than that of the TC treatment. Both treatments attained at their lowest weight on day 13 which was the first full day in Recovery (Figure 2B). Using simple arithmetic, the rate of LW loss in the TC group during Challenge (days 3 to 10) was approximately 3.1 kg∙steer^−1^∙d^−1^ whereas in Recovery (days 13 to 19), the rate of LW gain was 2.1 kg∙steer^−1^∙d^−1^.

### Rumen temperature

The daily mean RT for TC and FRTN animals is presented in Figure 2C. The RT of TC animals was elevated during Challenge compared with FRTN animals and remained so for 2 d into Recovery (*P* ≤ 0.0001). There was a transient rise of 0.11 to 0.26 °C in the RT of the FRTN steers relative days 1 and 2. The RT of the TC animals rose during Pre-Challenge and was slightly greater on days 3 and 4 (Pre-Challenge) than FRTN animals (*P* ≤ 0.003). It is likely that the difference between treatments reflected the slightly higher THI conditions experienced by the TC group during Pre-Challenge (see Figure 1). During Challenge, the RT of the TC animals rose and was greater than FRTN animals for days 5 to 13 (*P* ≤ 0.0001); it peaked on day 5 (40.65 ± 0.09 °C) in comparison to FRTN animals (39.77 ± 0.09 °C; *P* ≤ 0.0001; Figure 2C). Both groups experienced a gradual decline in mean daily RT during Challenge. The daily mean RT of the TC group was 40.65 °C for days 5 to 7; and recorded 40.38 °C on day 11, a reduction of 0.27 °C. From a high of 39.80 °C on day 4, the daily mean RT of the FRTN group declined to 39.37 °C by day 11, a reduction of 0.43 °C.

In Recovery, the RT of the TC animals fell rapidly and then stabilized over days 14 to 18 (at 39.54 to 39.59 °C), approximately 0.23 °C below that of Pre-Challenge days 1 and 2. The RT of the FRTN treatment continued its gradual descent reaching a minimum on day 14 (39.20 °C). Days 15 to 18 saw increased RT for this group, so that by days 16 to 18, there was no significant difference with the mean RT of the TC group (Figure 2C). It is interesting to note that the RT of the FRTN group in the last days of Recovery were about 0.25 °C lower than the Pre-Challenge days 1 to 3 also.

### Respiration rate and panting score

The mean daily RR of the TC group was greater (*P* ≤ 0.0001) compared with the FRTN animals during Challenge (days 5 to 11) and remained so till day 15 (*P =* 0.040; Figure 2D). The mean daily RR of the FRTN group gradually fell during Challenge, falling from 79 bpm on day 6 to 63 bpm on day 11; and in Recovery the FRTN daily mean RR continued to fall arriving at a minimum of 59 bpm on day 13. In contrast, with the onset of increased heat load (Challenge) the daily mean RR for the TC group rose rapidly and was at a plateau of 109 to 115 bpm on days 5 to 11. The rate of rise in RR during transition was determined to be 4.6 bpm/°C TA or 4.4 bpm/THI unit. (Taking Pre-Challenge (days 1 to 3) daily means of TA = 22.9 °C, THI = 70.1, and RR = 78.8 bpm versus mean daily TA, THI and RR during Challenge as 30.2 °C, 77.7 and 112.3 bpm, respectively). During Recovery, RR fell quickly and had stabilized by day 15. In the final days of Recovery (days 15 to 18), the mean daily RR of both groups converged (*P ≥* 0.14) but were 9 to 13 bpm lower than the Pre-Challenge means.

Panting Score (PS) can be used as a visual proxy for RR (Gaughan et al., 2010). Daily mean PS was impacted by treatment and period (Figure 2E), and closely followed daily RR mean (Figure 2D). The overall trend for the FRTN group was a gradual decline over the 18 d in the CCR although the minimum daily mean PS was achieved on day 8 (mean PS = 1.05 ± 0.05). The TC group experienced elevated and stable daily mean PS (overall average PS = 1.88 ± 0.02) compared with that of the FRTN group during Challenge (*P ≤* 0.0001). In Recovery, the PS of the TC group fell rapidly, and the PS means did not differ between the groups by days 14 and 15 (Figure 2E). Similar to the mean daily RR, in Recovery the mean daily PS of both groups was less than the Pre-Challenge means.

### Surface temperature

Shoulder ST gave the highest daily mean ST; however, head ST showed the largest and most consistent difference between TC and FRTN animals during Challenge (Figure 2F, Supplementary Figure S1). The TC group’s daily mean head ST were greater than those of the FRTN group from days 3 to 13; that is, during the last days of Pre-Challenge, throughout Challenge and the first days of Recovery. In the transition from Pre-Challenge to Challenge, the head ST of the TC group rose 4.1 °C by day 5. While the TC mean daily head ST was reasonably stable during Challenge, it was greater on days 9 to 11; the highest head ST occurred on day 10 at 31.24 °C, which was 8.33 °C greater than the corresponding head ST of the FRTN group (*P <* 0.0001). Mean daily head ST of the TC group fell quickly in Recovery and stabilized. For each group, the mean daily head ST in Recovery was similar to the Pre-Challenge means.

### Water consumption

The WC of the TC cattle increased (*P ≤* 0.003) during Challenge compared with FRTN animals (Figure 2G), whereas the WC of the FRTN animals decreased over the same period. The WC of both treatment groups was greater on day 1 relative all other days in Pre-Challenge due to training the animals to utilize the drinker; training was completed at the end of day 1. The greatest WC during Challenge occurred on day 9 with the TC animals consuming 56.63 ± 2.95 L∙steer^−1^∙d^−1^ (Figure 2G). The WC of the TC animals appeared to plateau over days 7 to 11 at a rate of 52 to 57 L∙steer^−1^∙d^−1^ while the WC of the FRTN group maintained its downward trajectory from 45 to 35 L∙steer^−1^∙d^−1^ during those days. In Recovery, both groups consumed about 40 L water∙steer^−1^∙d^−1^ which was less than the Pre-Challenge WC means.

### Relationships between daily mean RT and other measures

**Figure 3.**
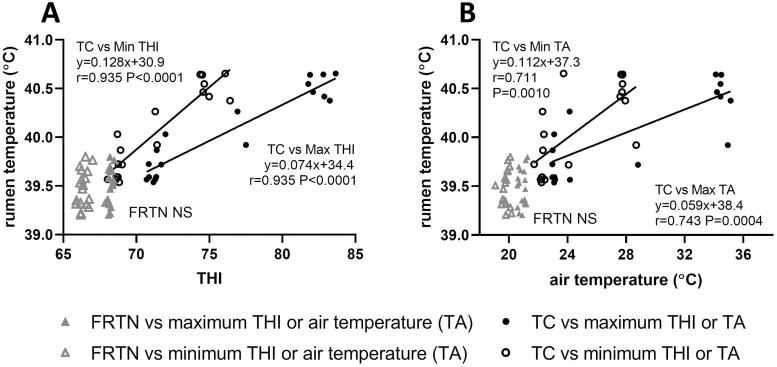
The relationships of mean daily rumen temperature of the thermally challenged (TC) and feed restricted thermoneutral (FRTN) groups to (A) daily minimum and maximum temperature-humidity index (THI) and (B) daily minimum and maximum air temperature TA). The line-of-best fit and equation are given along with Pearson correlation *r* and level of significance. NS, not significant.

The FRTN group experienced a daily mean RT range of 39.22 to 39.80 °C, whereas the range of the daily mean RT of the TC group was 39.54 to 40.65 °C. The relationships described below can only pertain to these ranges. Daily maximum and minimum THI strongly correlated with the daily mean RT of the TC group (*r* = 0.943, *P <*0.0001; and *r* = 0.935, *P <*0.0001, respectively; Figure 3A). For the THI ranges imposed on the TC group, the linear relationships predicted a 0.074 °C rise in RT per increment in daily maximum THI, and an increase of 0.128 °C RT/increment in daily minimum THI. TA also correlated well with the mean daily RT of the TC group with Pearson correlations *r* of 0.743 (*P =* 0.0004) and 0.710 (*P =* 0.0010) with daily maximum and minimum TA, respectively (Figure 3B). Within the narrow climatic range experienced by the FRTN group, no relationships with THI or TA were discoverable (Figure 3A and B).

**Figure 4.**
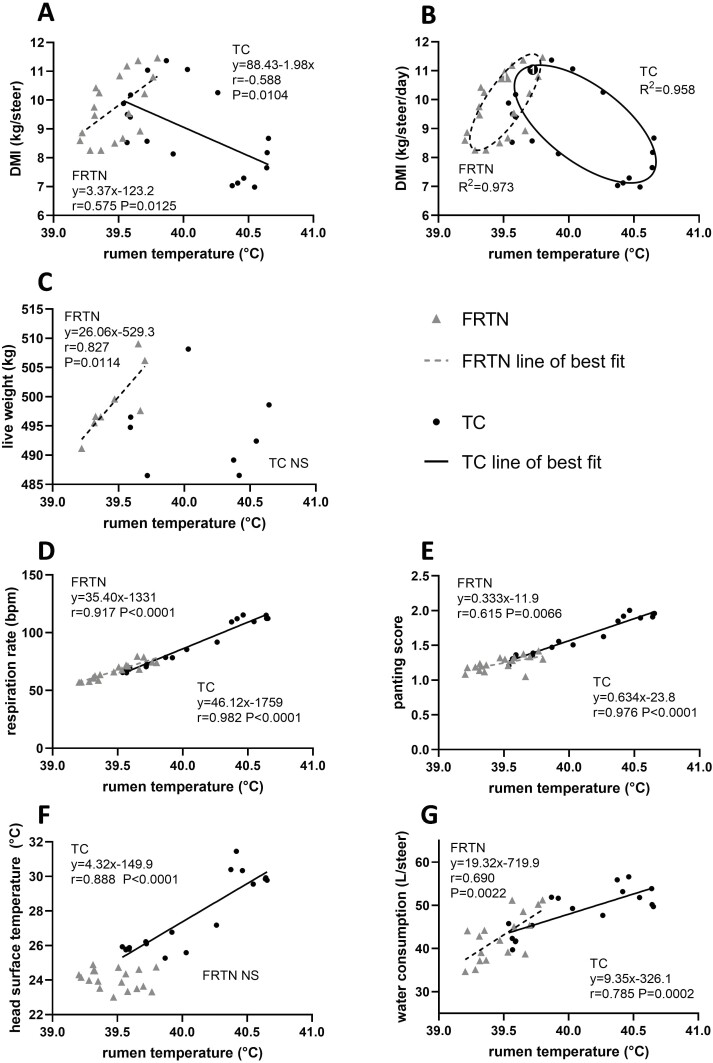
The relationships of animal performance and physiological variables with mean daily rumen temperature of the thermally challenged (TC) and feed restricted thermoneutral (FRTN) groups. (A) DMI with linear regression. (B) DMI with fitted ellipses. (C) Live weight. (D) Respiration rate. (F) Panting score. (F) Head surface temperature. (G) Water consumption. The line-of-best fit and linear equation are given along with Pearson correlation *r* and level of significance. *R*^2^, goodness of fit of the ellipses, NS, not significant.

The mean daily RT of the two treatment groups displayed opposing linear relationships with mean daily DMI (Figure 4A). The FRTN group had modest positive correlation between DMI and RT (*r* = 0.575; *P =* 0.0125), whereas a negative correlation was obtained for the TC group (*r* = −0.588; *P =* 0.0104). The overall rates of change in DMI per degree rise in RT according to linear relationships were 3.4 kg for the FRTN group, and −2.0 kg for the TC group. However, linear equations are manifestly poor approximations for the relationships between DMI and RT. Visual assessment discerned 2 opposing ellipsoid relationships, and indeed when fitting for elliptical equations, high correlations were obtained (*R*^2^ = 0.958 and 0.973 for the TC and FRTN groups, respectively; Figure 4B) (More detailed information on the characteristics of the ellipses and their equations are given in Supplementary Figure S2).

Both ellipses travel in a clockwise direction (day 1 for each ellipse is indicated in Figure 4B). For the TC group, days 1 and 2 are positioned at the top of the ellipse where DMI was high. The highest predicted mean DMI (11.12 kg∙steer^−1^∙d^−1^) occurred when RT was 39.82 °C. Day 7 (Challenge) occurred at the lower vertex and close to the predicted maximum daily mean RT of 40.65 °C (DMI: 8.2 kg∙steer^-−1^∙d^−1^). The lowest predicted DMI of 7.1 kg∙steer^−1^∙d^−1^ was at the base of the TC ellipse where RT was 40.33 °C. From this point, DMI increased as RT fell. Following the ellipse around to top vertex, the lowest RT, 39.24 °C, corresponded to a DMI of 10.1 kg∙steer^−1^∙d^−1^ (Figure 4B).

In the case of the FRTN group, DMI was the manipulated variable and RT the responding variable. The FRTN ellipse captured the small rise in RT during Pre-Challenge, so that the days 3 to 4 are positioned at the top vertex (Figure 4B). The lower vertex for the FRTN is circumscribed by days 12 to 14 (Recovery). The maximum daily mean DMI (11.31 kg∙steer^−1^∙d^−1^) occurred when RT was 39.67 °C; very similar to maximum DMI and associated RT of the TC group. At the FRTN group’s highest RT (39.77 °C), DMI had reduced slightly (10.72 kg∙steer^−1^∙d^−1^). As feed restriction was imposed, the lowest DMI (8.26 kg∙steer^−1^∙d^−1^) was associated with a RT of 39.35 °C. However, RT was at its lowest as DMI started to increase (39.24 °C; 8.93 kg∙steer^−1^∙d^−1^).

The relationships of RT with other parameters were linear or could not be defined. The LW of the FRTN group showed a strong positive correlation with RT (*r* = 0.827, *P =* 0.0114) and the linear relationship predicted a 26 kg increase in LW per 1 °C rise in RT (Figure 4C), with the qualification of the limited range in daily mean RT of this group. In contrast, there was no association between LW and RT in the TC group.

The FRTN group daily mean RT exhibited strong correlation with daily mean RR (*r* = 0.917, *P <* 0.0001), and the linear relationship predicted a ~35 bpm increase for each 1 °C increment in daily mean RT (Figure 4D). The TC group daily mean RT also strongly correlated with mean daily RR (*r* = 0.982, *P <* 0.0001), with a rate of change of approximately 46 bpm/°C RT. Not unexpectedly, relationships of mean daily PS of both groups to daily mean RT was similar to that of RR (Figure 4E), although the correlation between RT and PS for the FRTN group was moderate (*r* = 0.615; *P =* 0.0066). The rate of change of PS/°C RT was close to 0.33 and 0.63 for the FRTN and TC groups, respectively. Mean daily head ST of the FRTN group showed no relationship with RT whilst that of the TC group was strongly correlated (*r* = 0.888, *P <* 0.0001; Figure 4F) with a 4.3 °C rise in head ST/°C RT. Both groups showed moderate-to-strong positive correlations of mean daily WC with RT (Figure 4G) with the TC group having the stronger relationship (TC: *r* = 0.785, *P =* 0.0002 versus FRTN: *r* = 0.690, *P =* 0.0022). The apparent rate of increase in WC/°C RT in the FRTN group (at 19.3 L/°C) was double that of the TC group.

Visual inspection of Figure 4D, E, and G drew attention to the linear continuity in the distribution of data points contributed by the 2 treatment groups. When the daily mean RT, RR, PS, or WC from each group was combined, strong correlations and linear relationships were obtained with RT (RR: *r* = 0.978, *P <* 0.0001; PS: *r* = 0.958, *P <* 0.0001; WC: *r* = 0.785, *P <* 0.0001). Thus, the overall rates of increase of each parameter per 1 °C rise in RT were 41.5 bpm, 0.61 PS and 10.5 L apply and applied to both thermoneutral steers and steers with moderate heat load (see Supplementary Figure 3).

## Discussion

The study presented here is the first of a series of studies conducted on feedlot steers subjected to various thermal loads. One aim of the experiment was to understand how physiologically the feedlot steer responds to and *recovers from* a moderate multi-day heat load event in CCR when compared with its thermoneutral counterpart on a similar plane of nutrition. Secondly, the RT bolus has the potential to objectively inform feedlot operators in near real-time of the heat load of the cattle in their care, and the track dissipation of heat load in recovery. If this scenario is to become a reality, the behavior of RT and its relationships with other commonly used measures of performance and physiology of heat stressed cattle needs to be understood.

The feeding methodology in the current study applied a ‘pair offered’ regime, as opposed to a ‘pair feeding’ regime. It is generally understood that in a pair feeding regime the feed intake of the animal under challenge dictates the amount offered to the non-challenged counterpart. Necessarily, the pair feeding regime requires the pair fed animal be offered its quota one feed period behind that of its challenge pair. In the case of most thermal challenge experiments in ruminants, this equates to the intake of the thermally challenged (TC) animal setting the exact amount of feed offered to the thermoneutral (TN) pair the following day (for example, see O’Brien et al., 2010; Wheelock et al., 2010; Baumgard et al., 2011). However, there are animal health, welfare, and management implications in applying this regime to rapidly growing approximately 500 kg LW steers on high grain/concentrate diets. Sudden, large reductions or highly variable amounts of feed intake in beef cattle consuming 12 to 15 kg∙animal^−1^∙d^−1^ has consequences for rumen function and gut integrity and may induce stress through anxiety over change of routine.

Heat stress can invoke sudden large reduction in feed intake (≥ 50%) in feedlot animals (Beatty et al., 2006; Gaughan et al., 2010; Sullivan et al., 2011). Erratic feeding of feedlot cattle is known to impact rumen function and animal behaviors (reviewed by Schwartzkopf-Genswein et al., 2003). Zhang et al. (2013a) found increased ruminal pH in beef heifers subjected to 5 d feed restriction at 25%, 50%, and 75% of normal intake. At the highest level of feed restriction, there was evidence of compromised gut integrity. The same heifers were then fed ad libitum and rumen function monitored over the following 3 wk (Zhang et al., 2013b). Regardless of the previous level of feed restriction, ad libitum intake during recovery tended toward or caused ruminal acidosis. Recently, Rabaza et al. (2020) described increased pH range and breakdown of the ruminal pH circadian rhythm in feedlot steers that had feed withdrawn for 12 to 36 h. Moreover, sudden feed withdrawal for even short intervals induces a cortisol-mediated stress response (Marques et al., 2012, 2019). The feeding regime devised for this study was designed avoid the issues described above by maintaining control of the feed intake of the thermoneutral group during Challenge, and as such this group was feed restricted, and thus the termed feed restricted TN group (FRTN). The methodology applied a ‘pair-offered’ regime where the animals in each pair were offered the same amount of feed on the same day. The amount offered was based on the previous day’s intake of the TC animal plus an additional 20% feed.

### Responses to moderate heat load by the thermally challenged (TC) group

As anticipated, 6 full days of moderate heat load with a diurnal cycle delivering Danger and Alert levels of THI during Challenge impacted performance and physiology of the TC steers. In fact, the TC group responded immediately to the changing conditions on day 4, the transition day to Challenge conditions (days 5 to 11). The day 4 maximum THI was 77 whereas that of day 3 was 72; thus this small increase in daily maximum THI was sufficient to prompt a decrease in DMI, and increments in RT, RR, PS, and head ST. These observations support the concept of a THI threshold whereupon physiological and endocrine mechanisms are recruited to reduce endogenous heat production (rumen fermentation, metabolic activity), increase heat loss through convection by redirecting blood flow to respiratory mucosal and cutaneous surfaces, and evaporative cooling via the lungs (increased RR) and sweating (West, 2003; Collier et al., 2017).

Hahn et al. (1992) found that the core temperature of feeder cattle commenced an upward trajectory when TA ≥25 °C. Thresholds of similar TA or THI for feeder cattle have been determined by others (Lefcourt and Adams, 1996; Scharf et al., 2011a; Curtis et al., 2017). The rate of increase in core temperature in feeder steers when exposed to similar thermal loads as applied in the current study has ranged over 0.065 to 0.10 °C core temperature/°C TA and 0.075 to 0.10 °C core temperature/increment THI (Lefcourt and Adams, 1996; Brown-Brandl et al., 2003; O’Brien et al., 2010; Scharf et al., 2011a; Curtis et al., 2017). The steers in the current experiment produced a comparable rate of increase in core (rumen) temperature during the transition day (day 4) of 0.11 °C/°C in daily mean TA or 0.10 °C/daily mean THI unit. The sensitivity of core temperature to daily minimum THI or TA was evident with rate of increase in RT almost twice that of the rate of increase seen in response to daily maximum THI and TA.

Hahn et al. (1990) were among the first to note the simultaneous reduction of feed intake association of rising TA and core temperature in beef cattle. In the current experiment, the overall 0.66 °C rise in RT provoked by the moderate heat load was associated with a maximum 37.5% fall in daily DMI. From day 4, and once Challenge conditions were established, DMI fell by approximately 0.8 kg∙steer^−1^∙d^−1^ till day 8, but then stabilized in the last days of Challenge so that DMI was about 64% of the Pre-Challenge DMI. This magnitude of reduction in feed intake and its stabilization after 4 or so days at this level of thermal challenge accords with previous similar studies of feeder heifers and steers (Brown-Brandl et al., 2003; Beatty et al., 2006; Sullivan et al., 2011; Curtis et al., 2017).

The TC steers displayed a moderate negative relationship between DMI and RT during Challenge. Beatty et al. (2006) found a stronger correlation between RT and feed intake, whereas Curtis et al. (2017) found none. Having followed both groups through the 7 d of recovery in thermoneutral conditions, elliptical relationships were detected for DMI and RT. As far as can be gathered from the literature, this would appear to be the first description of such relationships in this context. Reduction in LW of the TC group at a rate of 3.1 kg∙steer^−1^∙d^−1^ was evident till day 10, whereupon it stabilized. There was an overall loss in LW of approximately 22 kg during Challenge, but no relationship was discovered between LW and RT. In contrast, the LW reduction of 18 kg by the FRTN group showed a strong correlation with RT.

Moderate thermal load in the TC group induced 34 bpm increase in daily mean RR, and an increase in daily mean PS of 0.5. RR and PS rose sharply with the onset of heat load also; the rate of increase in RR on transition day 4 was 4.4 bpm per THI increment and 4.6 bpm/°C TA, very close to the rate of 4.3 bpm/°C TA reported by Hahn (1999) for feeder cattle. Following the initial rapid rise in RR and PS on transition day, both parameters were constant for the 5 days of Challenge. The daily mean RR during Challenge was approximately 2-fold higher than the Pre-Challenge RR. This magnitude of increase and the stability in elevated RR once heat stress was established in steady day-to-day conditions (in CCR), was also observed by others in beef heifers, calves, and steers (Beatty et al., 2006; Scharf et al., 2010; O’Brien et al., 2010). Over the 18 d in the CCR, strong correlations and linear relationships of THI, RR, and PS with RT were evident, in agreement with previous studies in beef cattle (Beatty et al., 2006; Gaughan et al,. 2010; Scharf et al., 2011a,b; Gaughan and Mader, 2014). The rate of rise in RR and PS per 1 °C rise in RT was 46 bpm and 0.63 unit, respectively.

An initial response to increasing heat load is vasodilation of the capillary beds supporting the skin, with consequent increased blood flow to the skin to enable heat loss through convection (Finch, 1985). Increased temperature of the skin will promote warming of the body surface at most sites consequently enhancing dissipation of heat. Skin temperatures, as well as subdermal and subcutaneous temperatures have been shown to rise with increased thermal load and exhibit stronger correlation with TA and THI than with core temperature (Hahn et al., 1990: Mundia and Yamamoto, 1997; Scharf et al., 2010; Okada et al., 2013; Giro et al., 2019). Such observations suggest that STs are strongly influenced by environmental conditions.

Measurement of skin temperature requires ongoing removal of the hair at the site, and subdermal and subcutaneous temperature monitoring necessitates surgical insertion of devices. More recently infrared thermography (IRT) has been used to measure body ST at sites that are unshaved or unclipped (Church et al., 2014; Salles et al., 2016; Lees et al., 2018; Peng et al., 2019). This approach offers a rapid means of assessing increased heat load in production animals with the caveat that if STs are to be a proxy for core temperature, IRT must be performed free of interference from direct sunlight and wind (Okada et al., 2013; Montanholi et al., 2015). Under these conditions, STs are generally found to be significantly below core temperature and skin or subcutaneous temperature since the hair acts as a boundary layer between the skin and ambient conditions (Hoffmann et al., 2013; Church et al., 2014; Salles et al., 2016; Lees et al., 2018). While Montanholi et al. (2015) and Okada et al. (2013) showed good correspondence between ST and skin temperature at adjacent sites on the flank and shoulder, Giro et al. (2019) reported a correlation of 0.3 between subcutaneous temperature and ST at the base of the ear.

In the current study, head ST was found to give the most consistent measures compared to other body sites, and strong correlation to RT as RT approached 40 °C. These finding concur with those of Salles et al. (2016) and Peng et al. (2019) working with Jersey heifers and lactating Holstein cows. The forehead may be an ideal site for ST measurement in cattle breeds where the forehead is covered with short but dense hair that is less influence by seasonal changes (i.e., the growth of the winter coat).

The head ST of the TC steers rose rapidly with onset of heat load and the rate and magnitude of this response agreed with changes to trunk skin temperature by Scharf et al. (2010). During Challenge Head ST initially showed similar behavior as RT, RR, and PS, only to rise in the later days of Challenge. This behavior contrasted with RT which rose rapidly with onset of heat load, came to a plateau for the first 3 d of Challenge and then slowly declined. The phenomenon was also observed in steers subjected to increased heat load in CCR for 11 d by Brown-Brandl et al. (2003) but differed to Scharf et al. (2010) where trunk skin temperature fell after 4 d of heat load. Co-incidence in the delayed decline in RT with the late rise in head ST suggests that core temperature (as reported by RT) was somewhat offset by redirection of endogenous heat to the skin which presumably increased loss of heat to the environment. Furthermore, the changes in RT and head ST occurred just as WC was reached its highest levels over days 9 to 11. In combination, these observations and reduced stable DMI, support the generally accepted notion that it takes 3 to 4 d after the onset of a heat event for cattle to achieve a level of heat balance and for acclimation to begin (Hahn, 1999).

The TC group had elevated WC during increased thermal load, rising at a rate of 9 to 10 L/°C rise in RT. The levels of WC in the current experiment are very close to those reported by Gaughan et al. (2010) for lot fed shaded Angus steers of similar LW during hot conditions. It is well documented that WC increases in hot conditions (Beatty et al., 2006; Arias and Mader, 2011; Sullivan et al., 2011; Ahlberg et al., 2018). Increases in WC are an attempt to reduce heat load through peripheral vasodilation, perspiration, and water evaporation (Ahlberg et al., 2018). The re-purposing of WC to contribute to cooling mechanisms is illustrated by the diminished (or completely lost) relationships of WC with DMI and LW seen under thermoneutral conditions (unpublished data).

### Responses to underfeeding (the FRTN group)

By design, the DMI and LW of the FRTN group showed similar trajectories during Challenge as the TC group. Despite the slightly higher DMI, the LW of the 2 groups were very comparable. As feed restriction was implemented, the RT and RR tracked a steady descent. PS and WC showed this trend also albeit with greater variability. Head ST appeared unaffected by reduced DMI. The fall in RT and RR in the FRTN group can be expected given reduced organ size (especially of the liver), metabolic heat production, and requirement for oxygen in response to feed restriction (Richmond et al., 1988; Burrin et al., 1989; 1990; Eisemann and Nienaber, 1990; Nozière et al., 1999; Ekpe and Christopherson, 2000; Freetly et al., 2006).

Notwithstanding the constant thermoneutral conditions imposed on the FRTN group, these animals experienced a mean rise of 0.19 °C in RT over days 3 to 6. This transient elevation in RT may reflect a stress induced hyperthermia (SIH), a response observed in numerous animal species, but best studied in rat models (reviewed by Oka, 2018). Given that the steers in this study had limited exposure to handling and the indoor environment, it is conceivable that being housed (enclosed) in the CCR, and the altered routine may have provoked a SIH. Pedernera-Romano et al. (2010) reported SIH in sheep subjected to new environments, and it has been reported that housing novice cattle in research facilities can induce a stress response (Lomborg et al., 2008). It is acknowledged that the current experiment may not have allowed sufficient time for familiarization with the new surroundings and the new management routine before the Pre-Challenge period. However, the potential SIH was a transient response.

After day 6, the FRTN RT slowly decreased so that by day 11 RT had fallen 0.26 °C. Moreover, RR gradually decreased at the same time. These effects are most likely due to the reduced DMI and the consequent reduction of endogenous heat production from metabolism (Burrin et al., 1989; 1990; Eisemann and Nienaber, 1990; Ekpe and Christopherson, 2000) and rumen fermentation (Cole and Hutchinson, 1981; 1985; Soto-Navarro et al., 2000; Zhang et al., 2013a). Interestingly, inspection of the rate of decline in RT in the TC group over days 7 to 11 showed a similar reduction (0.27 °C). Thus, it appears that the voluntary reduction of DMI was the major contributor to the lowering daily mean RT over time in the TC group as there was no concurrent increase in RR. The interaction between the level of DMI, thus metabolic and rumen fermentative activities, and core temperature during periods of high heat load is critical.

### Recovery after moderate heat load or underfeeding

The transition from Challenge to Recovery conditions was as rapid as that for Pre-Challenge to Challenge conditions, thus the transitions between conditions were symmetrical. However, this symmetry was not exhibited by the performance measures in either group. DMI rose gradually for the TC group during the 7 d of Recovery but did not return to Pre-Challenge DMI. Beatty et al. (2006) also noted a slow rise in feed intake in beef heifers following thermal challenge in CCR. In the current experiment, DMI for the TC group in Recovery showed average daily increments of 0.4 kg∙steer^−1^∙d^−1^, whereas it is rate of decrease during Challenge was approximately 0.58 kg∙steer^−1^∙d^−1^. Likewise, LW had not returned to its Pre-Challenge weight and the rate of LW gain in Recovery was about two-thirds the rate of loss in Challenge. Due to restricted feeding based on the DMI of the TC group, the FRTN group had very similar reverses of DMI and LW. In contrast, most of the physiological responses provoked in the TC group by the increased thermal load adjusted rapidly as conditions transitioned between Challenge and Recovery. It is worth noting that the TC steers required 2 full days after the abrupt cessation of moderate heat load to completely dissipate their accumulated heat load. In parallel, the FRTN group terminated the downward trajectories of most parameters. The most striking aspect of Recovery was the lowered RT, RR, PS, and WC in both groups compared with Pre-Challenge, suggesting a state of lowered metabolism despite increasing DMI.

For the 39.2 to 40.6 °C range in daily mean RT explored in this study, thresholds of THI with RT and RR were evident. However, the linear relationships obtained for mean daily RR, PS, head ST, and WC with mean daily RT suggested a highly co-ordinated responses between RT and use of respiration and skin as means of heat loss via evaporation (reviewed by Bianca, 1965; Richards, 1973). Across all animals and conditions, every 0.5 °C increment in mean daily RT was associated with increases of approximately 20 bpm in mean daily RR, 0.3 in mean daily PS, 2.35 °C in mean daily head ST, and approximately 5 L in mean daily WC.

## Conclusion

Performance-wise and physiologically, the TC steers when subjected to moderate thermal load responded in similar manner as those described for beef cattle in comparable conditions. With frequent measurement during Challenge and Recovery, new aspects of relationships between them were revealed for both treatment groups. The most novel discovery was the elliptical relationship of RT with DMI in both the TC and FRTN groups which exemplified non-linear homeorhesis processes of physiological adjustment to a new state appropriate to the new conditions and then its reversal as Recovery conditions were imposed. The elliptical relationship arises from an inertia move to the ‘new’ state and the 3 to 5 d to acquire stability in this state. It is noteworthy that the DMI versus RT ellipses were underpinned by linear relationships of RT with other physiological parameters. That is, when pooling the daily means obtained over the 18 d for both treatment group, the linear relationships of RT with RR, PS, and WC were maintained. Therefore, over a mean daily RT range of 39.2 to 40.6 °C in beef steers on a similar plane of nutrition, there were consistent rates of response by these physiological parameters.

Perhaps novel to the current study is the observed lowering of mean daily RT, RR, and PS in the FRTN group during Challenge when feed restriction was imposed on the FRTN group; such observations had not been previously described in earlier studies contrasting pair-fed thermoneutral cattle with heat stressed animals. Furthermore, the transient stress-induced hyperthermia detected in this group has not been reported in other studies, and while this may reflect few days of familiarization to housing and handling during Pre-Challenge, it is possible that similar responses were overlooked in other studies. On the whole, and reassuringly, this study has demonstrated that the 5 d of moderate heat load, typical of a Queensland summer, was well within the homeorhetic capacity of feedlot cattle.

## Supplementary Material

skac127_suppl_Supplementary_Material

## Abbreviations

CCR: climate-controlled rooms
FRTN: feed restricted thermoneutral
QASP: Queensland Animal Science Precinct
PS: panting score
RR: respiration rate
RT: rumen temperature
ST: surface temperature
TC: thermally challenged
TA: air temperature
THI: temperature-humidity index
WC: water consumption
