# Supplementary material for: Elliptical and linear relationships with rumen temperature support a homeorhetic trajectory for DMI during recovery of feedlot cattle exposed to moderate heat load
Source: J Anim Sci. 2022 Apr 13;100(5):skac127. doi: 10.1093/jas/skac127 (PMC9115906; doi:10.1093/jas/skac127)
Supplement: skac127_suppl_Supplementary_Material [file skac127_suppl_Supplementary_Material.docx]

**Supplementary Table 1.** The air temperature and temperature-humidity index conditions the steers experienced while in the paddock (before transfer to the feedlot pen) and in the 40-d feedlot period before entering the climate-controlled rooms. TA: air temperature, THI: temperature-humidity index

| Cohort | 1 | 2 | 3 |
| --- | --- | --- | --- |
| Paddock weather conditions |  |  |  |
| Days in paddock (till feedlot entry) | 73 | 87 | 109 |
| Daily mean maximum TA (± SD), °C | 25.0 ± 3.0 | 25.0 ± 3.2 | 23.6 ± 3.4 |
| Daily mean minimum TA (± SD), °C | 12.4 ± 2.9 | 11.8 ± 3.3 | 10.8 ± 3.9 |
| Daily mean maximum THI (± SD) | 71.7 ± 3.6 | 71.0 ± 3.9 | 69.7 ± 4.4 |
| Daily mean minimum THI (± SD) | 54.8 ± 4.5 | 53.7 ± 5.3 | 51.9 ± 6.3 |
| Overall maximum TA, °C | 31.6 | 31.6 | 31.6 |
| Overall minimum TA, °C | 5.5 | 1.2 | 0.7 |
| Overall maximum THI | 80.4 | 80.4 | 80.4 |
| Overall minimum THI | 46.6 | 35.5 | 38.2 |
| Feedlot weather conditions | | | |
| Daily mean maximum TA (± SD), °C | 20.8 ± 2.1 | 21.3 ± 2.4 | 22.0 ± 2.4 |
| Daily mean minimum TA (± SD), °C | 7.3 ± 3.0 | 6.5 ± 2.6 | 8.3 ± 2.8 |
| Daily mean maximum THI (± SD) | 65.9 ± 2.7 | 66.1 ± 2.6 | 67.1 ± 2.4 |
| Daily mean minimum THI (± SD) | 46.3 ± 5.1 | 44.9 ± 4.4 | 47.8 ± 4.7 |
| Overall maximum TA, °C | 25.3 | 27.9 | 27.9 |
| Overall minimum TA, °C | 0.7 | 0.7 | 2.6 |
| Overall maximum THI | 72.1 | 72.4 | 72.4 |
| Overall minimum THI | 35.2 | 35.2 | 38.5 |

**Supplementary Table 2.** Climatic conditions across entire period in the climate-controlled rooms for the TC treatment. The daily minimum (min), maximum (max) and mean ambient temperatures (TA), relative humidity (RH) and temperature-humidity index (THI) are listed below: acclimation – d 0; Pre-Challenge, d 1 to 4; Challenge, d 5 to 11 (boxed and in bold); Recovery, d 12 to 18

| day | min TA, ºC | max TA, ºC | mean TA, ºC | min RH, % | max RH, % | mean RH, % | min  THI | max  THI | mean THI |
| --- | --- | --- | --- | --- | --- | --- | --- | --- | --- |
| 0 | 21.73 | 22.97 | 22.48 | 29.30 | 65.65 | 52.79 | 65.93 | 70.24 | 68.71 |
| 1 | 22.33 | 23.00 | 22.68 | 57.14 | 78.75 | 64.62 | 68.76 | 70.85 | 69.81 |
| 2 | 22.40 | 23.09 | 22.70 | 59.93 | 77.15 | 64.69 | 68.92 | 71.39 | 69.83 |
| 3 | 22.30 | 24.15 | 23.32 | 58.98 | 77.81 | 63.93 | 68.71 | 71.99 | 70.60 |
| 4 | 23.74 | 27.81 | 25.53 | 53.08 | 73.56 | 61.74 | 71.29 | 76.93 | 73.51 |
| 5 | **27.66** | **34.13** | **29.92** | **44.42** | **66.84** | **57.85** | **76.08** | **83.65** | **78.90** |
| 6 | **27.76** | **34.50** | **29.89** | **31.05** | **66.02** | **47.71** | **74.37** | **82.81** | **77.27** |
| 7 | **27.76** | **34.45** | **29.90** | **35.27** | **52.02** | **44.43** | **74.52** | **81.89** | **76.84** |
| 8 | **27.73** | **34.21** | **29.89** | **35.19** | **52.73** | **45.02** | **74.65** | **81.77** | **76.97** |
| 9 | **27.61** | **34.45** | **30.11** | **33.56** | **53.01** | **45.30** | **74.57** | **82.10** | **77.31** |
| 10 | **27.96** | **35.14** | **30.65** | **35.20** | **54.32** | **45.09** | **74.98** | **82.87** | **77.90** |
| 11 | **28.70** | **34.95** | **30.87** | **40.00** | **58.42** | **49.99** | **76.42** | **83.25** | **79.04** |
| 12 | 24.08 | 28.82 | 25.28 | 52.82 | 74.85 | 61.03 | 71.42 | 77.50 | 73.10 |
| 13 | 22.45 | 24.18 | 22.84 | 57.75 | 73.61 | 62.51 | 69.03 | 71.70 | 69.85 |
| 14 | 22.25 | 22.97 | 22.62 | 56.85 | 78.41 | 63.14 | 68.59 | 71.71 | 69.57 |
| 15 | 22.23 | 23.09 | 22.67 | 57.58 | 73.08 | 63.51 | 68.74 | 70.87 | 69.64 |
| 16 | 22.28 | 23.12 | 22.68 | 58.60 | 82.75 | 64.32 | 68.83 | 71.17 | 69.69 |
| 17 | 22.16 | 23.17 | 22.55 | 59.20 | 77.77 | 63.70 | 68.63 | 71.36 | 69.45 |
| 18 | 21.87 | 22.93 | 22.43 | 57.50 | 78.50 | 62.62 | 68.03 | 71.29 | 69.18 |

**Supplementary Figure 1.** Daily mean (± SEM) surface temperatures of all four sites (head, shoulder, leg and rump) during the 18 d in the climate-controlled rooms. A. The surface temperatures of the feed restricted thermoneutral (FRTN) group. B. The surface temperatures of the thermally challenged (TC) group. The boxed area indicates the Challenge period.

**Supplementary Figure 2.** Attributes of the ellipses fitted for the relationships between mean daily rumen temperature (RT) and mean daily DMI for the treatment groups. A. Thermally challenged (TC) group. B. Feed restricted thermoneutral group (FRTN). The equations of the fitted ellipses use the following format: Ax^2^ + Bxy + Cy^2^ +Dx + Ey + F = 0.

**Supplementary Figure 3.** Linear relationships with daily mean rumen temperature of the pooled daily means of the of the thermally challenged (TC) and feed restricted thermoneutral (FRTN) groups during the 18 d in the climate-controlled rooms. A. Respiration rate. B. Panting score. C. Head surface temperature. D. Water consumption. The line-of-best fit and equation are given along with Pearson correlation r and level of significance.
